# Supplementary material for: Peroxisomal core structures segregate diverse metabolic pathways
Source: Nat Commun. 2025 Feb 20;16:1802. doi: 10.1038/s41467-025-57053-9 (PMC11842775; doi:10.1038/s41467-025-57053-9)
Supplement: Supplementary file 1 — Supplementary Information [file 41467_2025_57053_MOESM1_ESM.pdf]

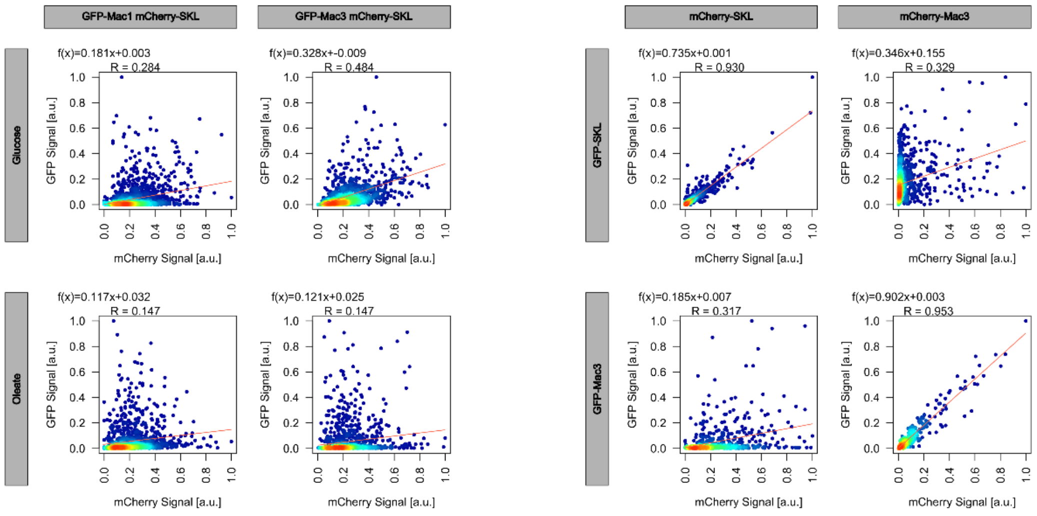

Figure S1 Examples of Plots generated for peroxisome quantification and co-localization analysis.
Representative plots showing the fluorescence signals of both fluorescent proteins in individual peroxisomes (dots).

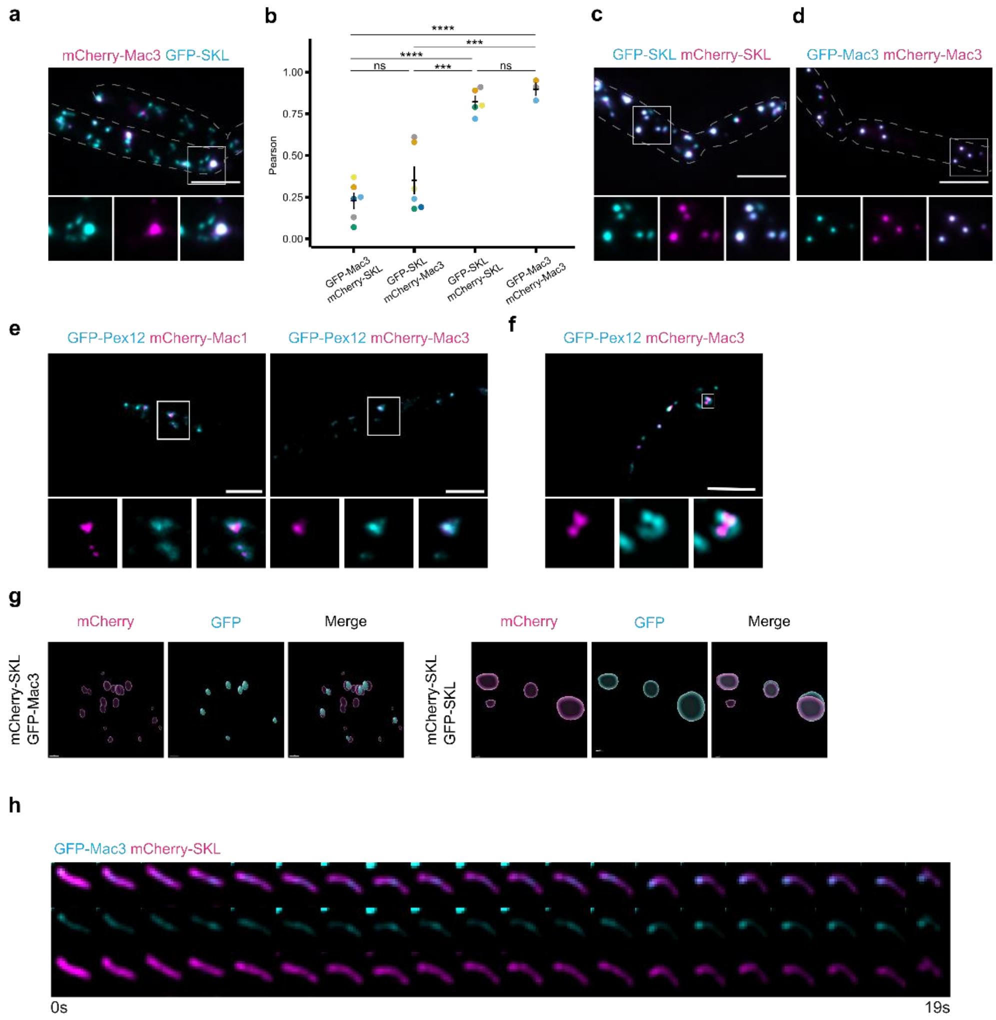

Figure S2 Mac3 localizes in peroxisome subdomains and subpopulations.
a mCherry-Mac3 and GFP-SKL were co-expressed and visualized by epifluorescence microscopy. Full image shown as overlay of the green and red channel. For insets single channels and merged channels

are depicted.**b** Quantification shows Pearson's correlation coefficients of GFP and mCherry signals for indicated strains. Plotted are the means of three replicates. Different colors denote single experiments. Center line, mean; error bars; standard error of the mean. Source data are provided in the source data file. *** refers to a p-value lower than or equal to 0.001, **** refers to a p-value lower or equal to 0.0001 and n.s. refers to a p-value greater than 0.05. Exact p-values are provided in the source data file. Error bars on the data points denote the regression error, while the error bars on the column denote standard deviation of the mean. Significance was assessed with a one-way ANOVA with post-hoc Tukey HSD Test. **c and d** GFP-SKL and mCherry-SKL or GFP-Mac3 and mCherry-Mac3 were co-expressed and representative epifluorescence images are shown. Organization of pictures as described above. **e** *U. maydis* strains expressing N-terminally mCherry-tagged versions of Mac3 or Mac1 (magenta) and the peroxisomal membrane protein Pex12 tagged with GFP (cyan) were inspected by epifluorescence microscopy. Full images are shown as overlays of two channels. For insets single channels and merged channels are depicted. **f** *U. maydis* strains expressing GFP-tagged Pex12 (cyan) and mCherry-Mac3 (magenta) were inspected by SIM. Organization of pictures as above. **g** 3D-reconstruction in x-z-projection of GFP-Mac1, GFP-Mac3 and GFP-SKL (cyan) containing peroxisomes, mCherry-SKL (magenta). Scale bars: 0.5 µm **h** Time lapse imaging related to Movie 1. Epifluorescence imaging of GFP-Mac3 and mCherry-SKL. One frame per second was recorded. Shown are overlays of the mCherry and GFP signals.

All GFP signals are shown in cyan and mCherry signals in magenta. Scale bars: 5 µm if not noted otherwise.

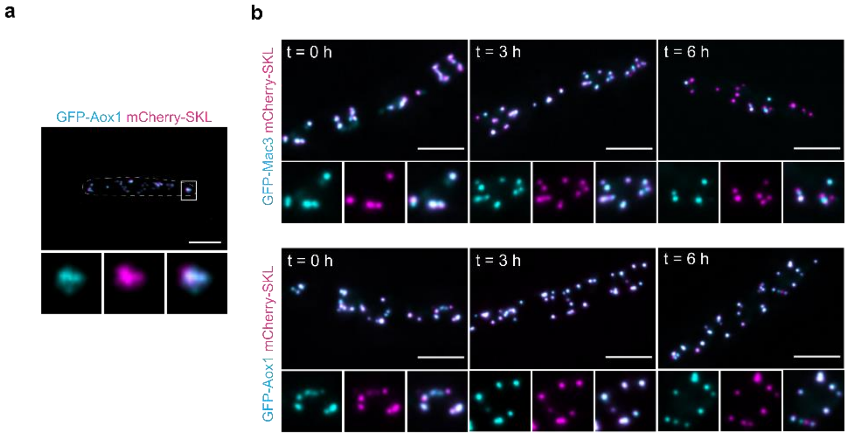

Figure S3 Subpopulations containing Mac3 form after import.
a *U. maydis* strains expressing an N-terminally GFP-tagged version of Aox1 (cyan) and the peroxisomal marker protein mCherry-SKL (magenta) were inspected by epifluorescence microscopy. Full images are shown as overlays of the green and red channel. For insets single channels and merged channels are depicted. **b** Cells were analyzed at indicated time points after addition of glucose by epifluorescence microscopy. GFP-Mac3 (cyan) was compared to GFP-Aox1 (cyan) in strains containing mCherry-SKL (magenta). Full images are shown as overlays of the green and red channel. For insets single channels and merged channels are depicted. Quantifications of b are shown in Fig. 2.
Scale bars: 5 µm.

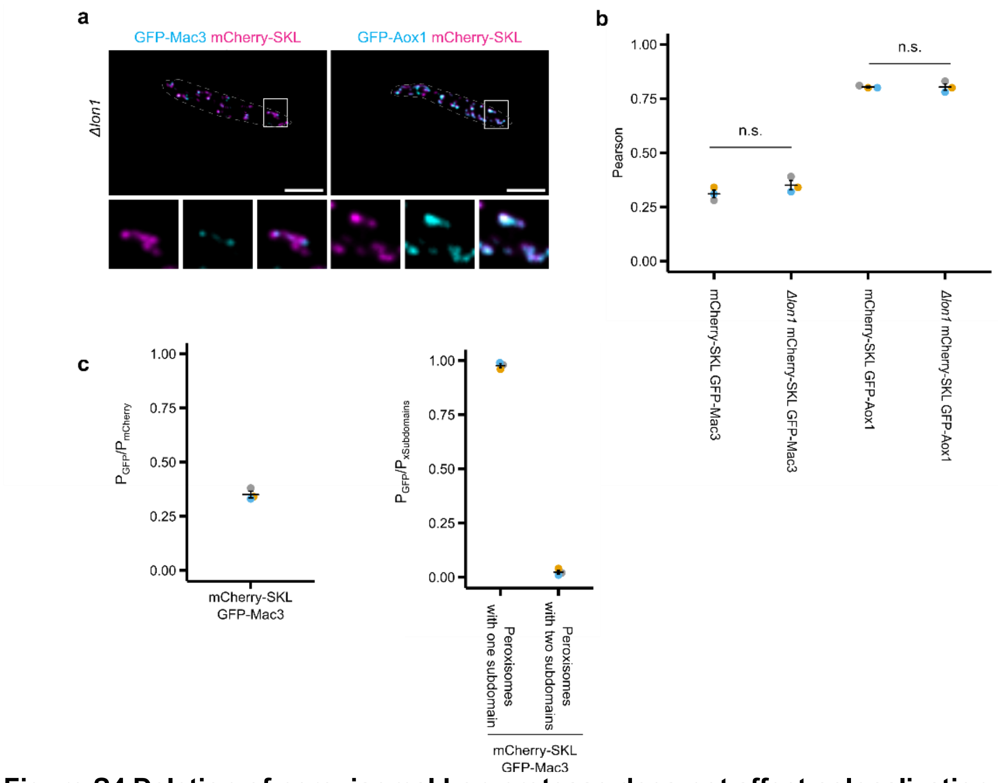

Figure S4 Deletion of peroxisomal Lon protease does not affect colocalization.
a *U. maydis* Lon deletion strains expressing N-terminally GFP-tagged versions of Mac3 or Aox1 (cyan) and the peroxisomal marker protein mCherry-SKL (magenta) were inspected by epifluorescence microscopy. Full images are shown as overlays of two channels. For insets single channels and merged channels are depicted. Scale bars: 5 µm. **b** Quantifications show Pearson's correlation coefficients of GFP and mCherry signals for indicated strains. Plotted are the means of three replicates. Different colors denote single experiments. Center line, mean; error bars; standard error of the mean. Significance was assessed with an unpaired, two-sided Student's t-test. Source data are provided in the source data file. n.s. refers to a p-value of 0.5122 (left) or 0.8451 (right). Error bars on the data points denote the regression error, while the error bars on the column denote standard deviation of the mean. Significance was assessed with a one-way ANOVA with post-hoc Tukey HSD Test. **c** Graph showing the relative number of peroxisomes containing a GFP-Mac3 (left) and how many subdomains they contain (right). Center line, mean; error bars; standard error of the mean.

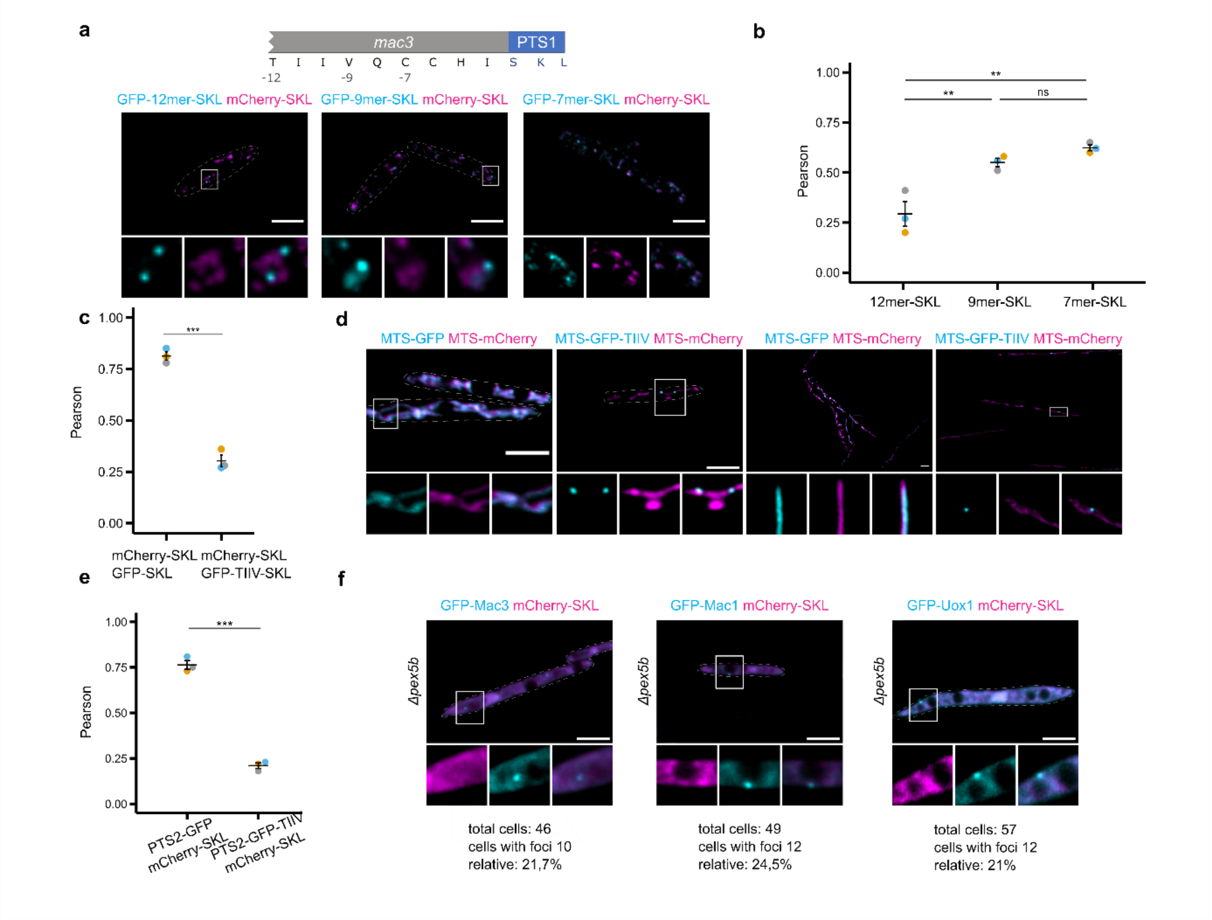

Figure S5 The amino acid motiv TIIV is necessary and sufficient to trigger focal accumulation of Mac3.

a Depiction of the 3'end of the *mac3* gene encoding the peroxisomal targeting signal PTS1. Please note that the unusual ASL motif had to be substituted by SKL to perform the subsequent truncation analysis as truncated fragment of the dodecamer did not allow full peroxisomal import, which perturbs colocalization analysis. Indicated fragments of the C-terminal dodecamer were fused to GFP (cyan) and analyzed with epifluorescence in strains also expressing mCherry-SKL (magenta). Full representative images are shown as overlays of the green and red channel. For insets single channels and merged channels are depicted. **b** Quantification of colocalization of experiments shown in a. Plotted are the means of three replicates. Different colors denote single experiments. Center line, mean; error bars; standard error of the mean. Source data are provided in the source data file. ** refers to a p-value of 0.00526 (lower) or 0.00144 (upper) and n.s. for a p-value greater of 0.373. Error bars on the data points denote the regression error, while the error bars on the column denote standard deviation of the mean. Significance was assessed with a one-way ANOVA with post-hoc Tukey HSD Test. **c** Quantification for experiments shown in Fig. 2e**.** Plotted are the means of three replicates. Different colors denote single experiments. Center line, mean; error bars; standard error of the mean. Significance was assessed with an unpaired, two-sided Student's t-test. Source data are provided in the source data file. *** refers to a p-value lower of 0.00012. Error bars on the data points denote the regression error, while the error bars on the column denote standard deviation of the mean. **d** GFP containing a mitochondrial targeting signal (MTS) either with or without an additional C-terminal TIIV motif was co-expressed with MTS-mCherry. Representative epifluorescence images are shown (left). Colocalization was also analyzed by SIM (right). **e** Quantification shows Pearson's correlation coefficients of GFP and mCherry signals for Fig. 2f. Plotted are the means of three replicates. Different colors denote single experiments. Center line, mean; error bars; standard error of the mean. Significance was assessed with an unpaired, two-sided Student's t-test. Source data are provided in the source data file. *** refers to a p-value lower of 0.00016. Error bars on the data points denote the regression error, while the error bars on the column denote standard deviation of the mean. Significance was assessed with an unpaired, two-sided Student's t-test. **f** *U. maydis* Pex5b deletion strains expressing N-terminally GFP-tagged versions of Mac3 (cyan), Mac1 or Uox1 (cyan) and the peroxisomal marker protein mCherry-SKL (magenta) were inspected by epifluorescence microscopy. Full images are shown as overlays of two channels. For insets single channels and merged channels are depicted. Scale bars: 5 µm.

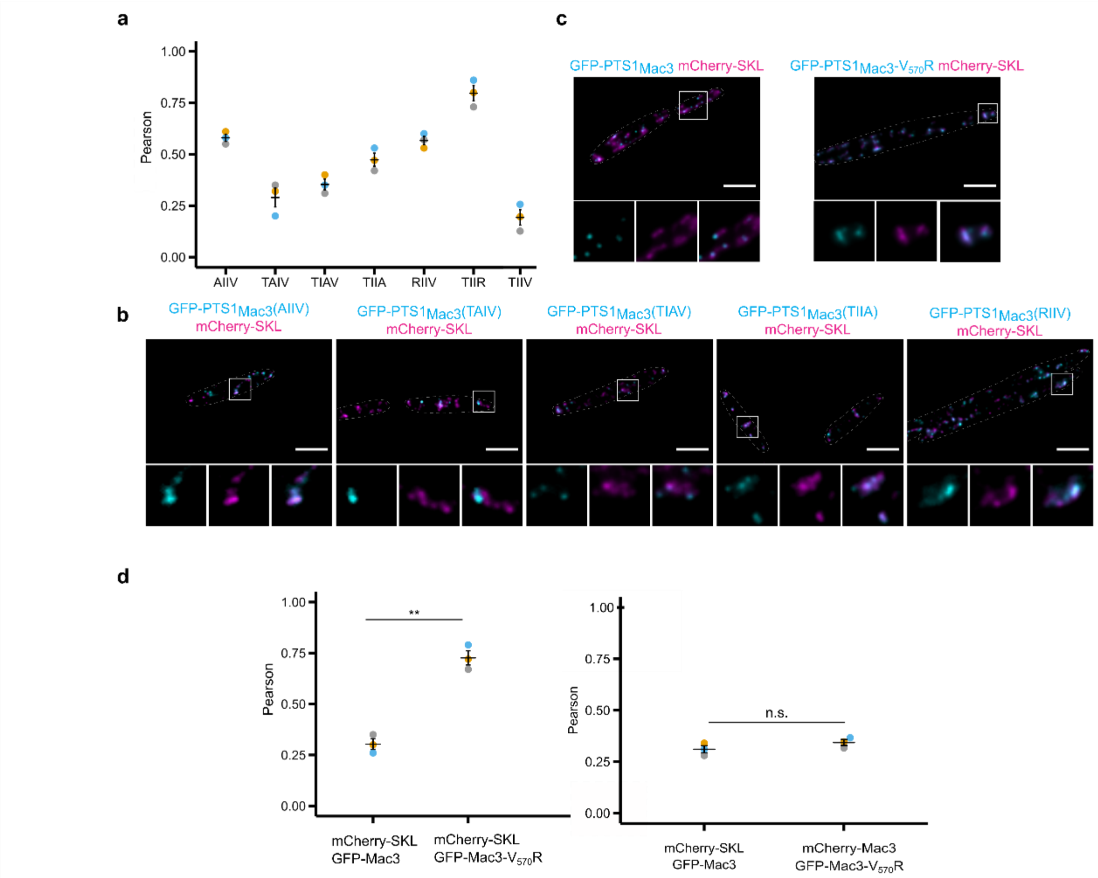

Figure S6 Analysis of the short amino acid motif TIIV and its derivatives.

a Quantification shows Pearson's correlation coefficients of GFP and mCherry signals for indicated mutants. Plotted are the means of three replicates. Different colors denote single experiments. Center line, mean; error bars; standard error of the mean. Error bars on the data points denote the regression error, while the error bars on the column denote standard deviation of the mean. **b** Representative epifluorescence images of each of the analyzed mutants. Full images are shown as overlays of the green and red channel. For insets single channels and merged channels are depicted. **c** Representative epifluorescence images of cells expressing GFP-PTS1$_{Mac3}$ and mCherry-SKL (left) or GFP-PTS1$_{Mac3}$V$_{570}$R and mCherry-SKL (right). Organization of pictures as above. **d** Quantifications of colocalization of experiments shown in Fig. 2g. Plotted are the means of three replicates. Different colors denote single experiments. Center line, mean; error bars; standard error of the mean. Significance was assessed with an unpaired, two-sided Student's t-test. Source data are provided in the source data file. ** refers to a p-value of 0.0023 and n.s. refers to a p-value of 0.0615. Error bars on the data points denote the regression error, while the error bars on the column denote standard deviation of the mean. Scale bars: 5 µm.

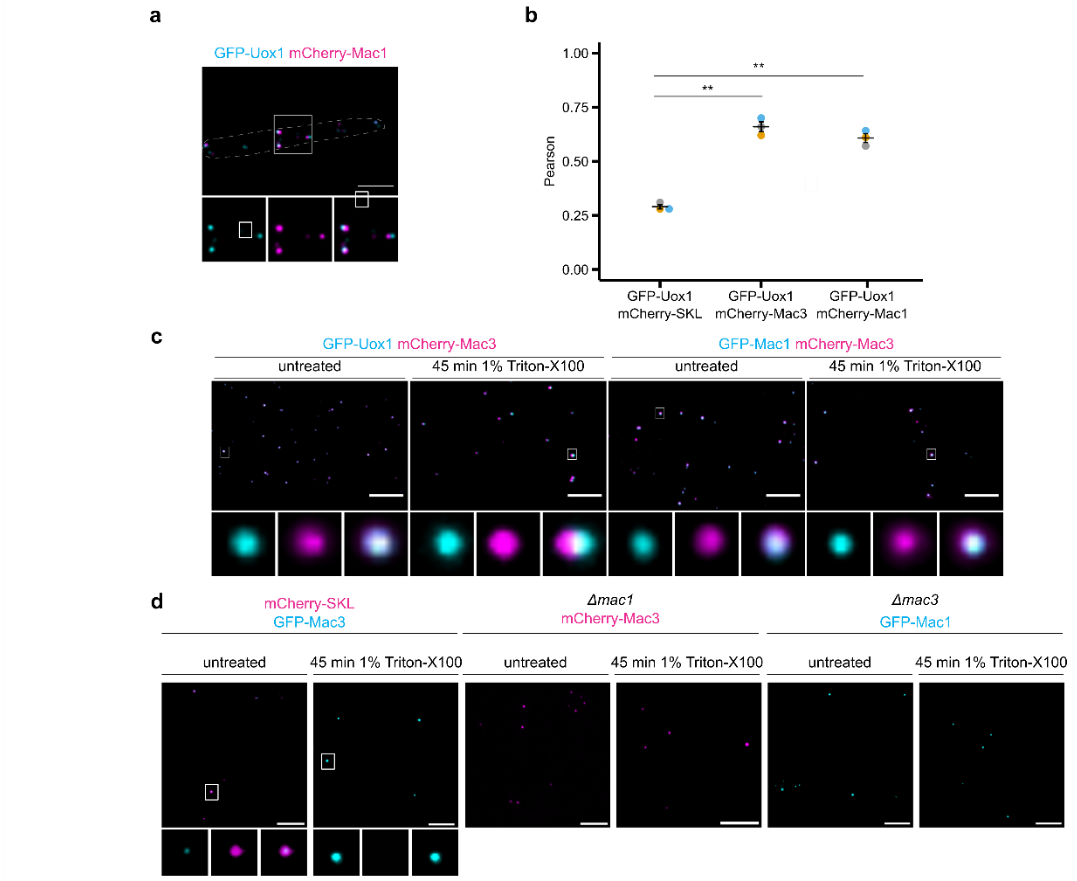

Figure S7 Colocalization of Uox1 and Mac3.
a Representative epifluorescence image a cell expressing GFP-Uox1 and mCherry-Mac1. Full images are shown as overlays of the green and red channel. For insets single channels and merged channels are depicted. **b** Quantifications of colocalization of experiments shown in a and Fig. 2a and b. Plotted are the means of three replicates. Different colors denote single experiments. Center line, mean; error bars; standard error of the mean. Significance was assessed with a one-way ANOVA with post-hoc Tukey HSD Test. Source data are provided in the source data file. ** refers to a p-value lower of 0.0046 (lower) or 0.0086 (upper). Error bars on the data points denote the regression error, while the error bars on the column denote standard deviation of the mean. **c** Crude organelle preparations of indicated strains were imaged by epifluorescence microscopy after incubation in lysis buffer supplemented with Triton X-100 (right). Preparations incubated in lysis buffer without Triton X-100 served as control (left). **d** Crude organelle preparation of indicated strains were analyzed by epifluorescence microscopy after incubation in lysis buffer (left) or in lysis buffer supplemented with Triton X-100 (right) for 45 min. Organization of pictures as described above.
Scale bars: 5 µm.

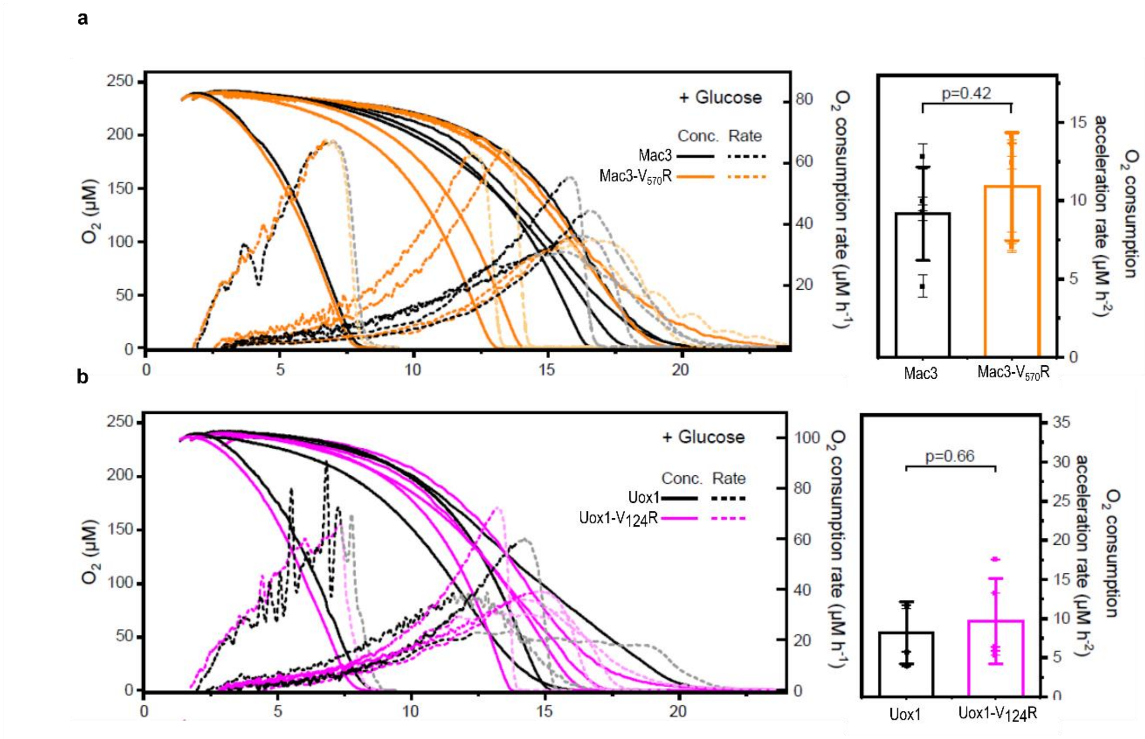

Figure S8 Variation of TIIV in Mac3 and Uox1 does not affect oxygen consumption rates upon incubation of cells in YNB-Glu.
Oxygen consumption of Mac3 and Mac3-V_{570}R **(a)** or Uox1 and Uox1-V_{124}R **(b)** was recorded over time (left). Data was plotted and analyzed as described for Figs. 4 and 5.

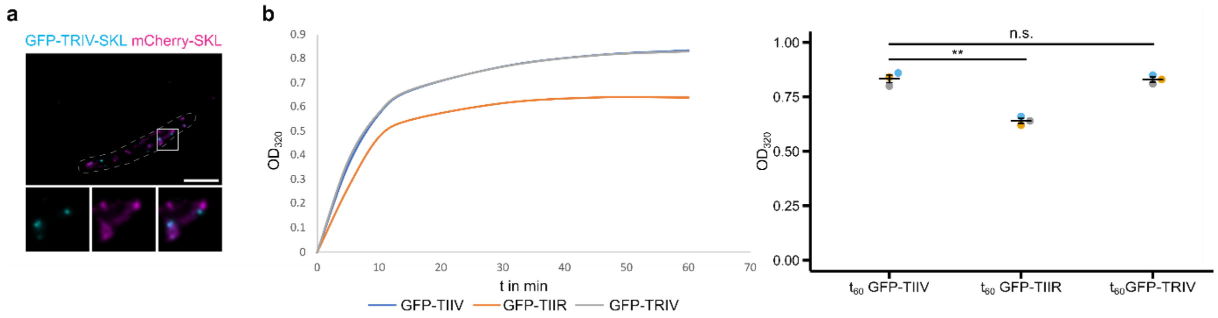

Figure S9 Analysis of the small motif TRIV from urate oxidase Uox1.
a Representative epifluorescence image of a cell expressing GFP-TRIV-SKL and mCherry-SKL. Full images are shown as overlays of the green and red channel. For insets single channels and merged channels are depicted. Scale bar: 5 µm. **b** Aggregation of GFP-TIIV, GFP-TIIR, and GFP-TRIV was measured over time (left). Plotted are the means of three replicates for each protein (left) and a comparison at 60 min (right). Plotted are the means of three replicates. Different colors denote single experiments. Center line, mean; error bars; standard error of the mean. Significance was assessed with a one-way ANOVA with post-hoc Tukey HSD Test. Source data are provided in the source data file. ** refers to a p-value of 0.0082 and n.s. to a p-value greater of 0.882. Error bars on the data points denote the regression error, while the error bars on the column denote standard deviation of the mean.

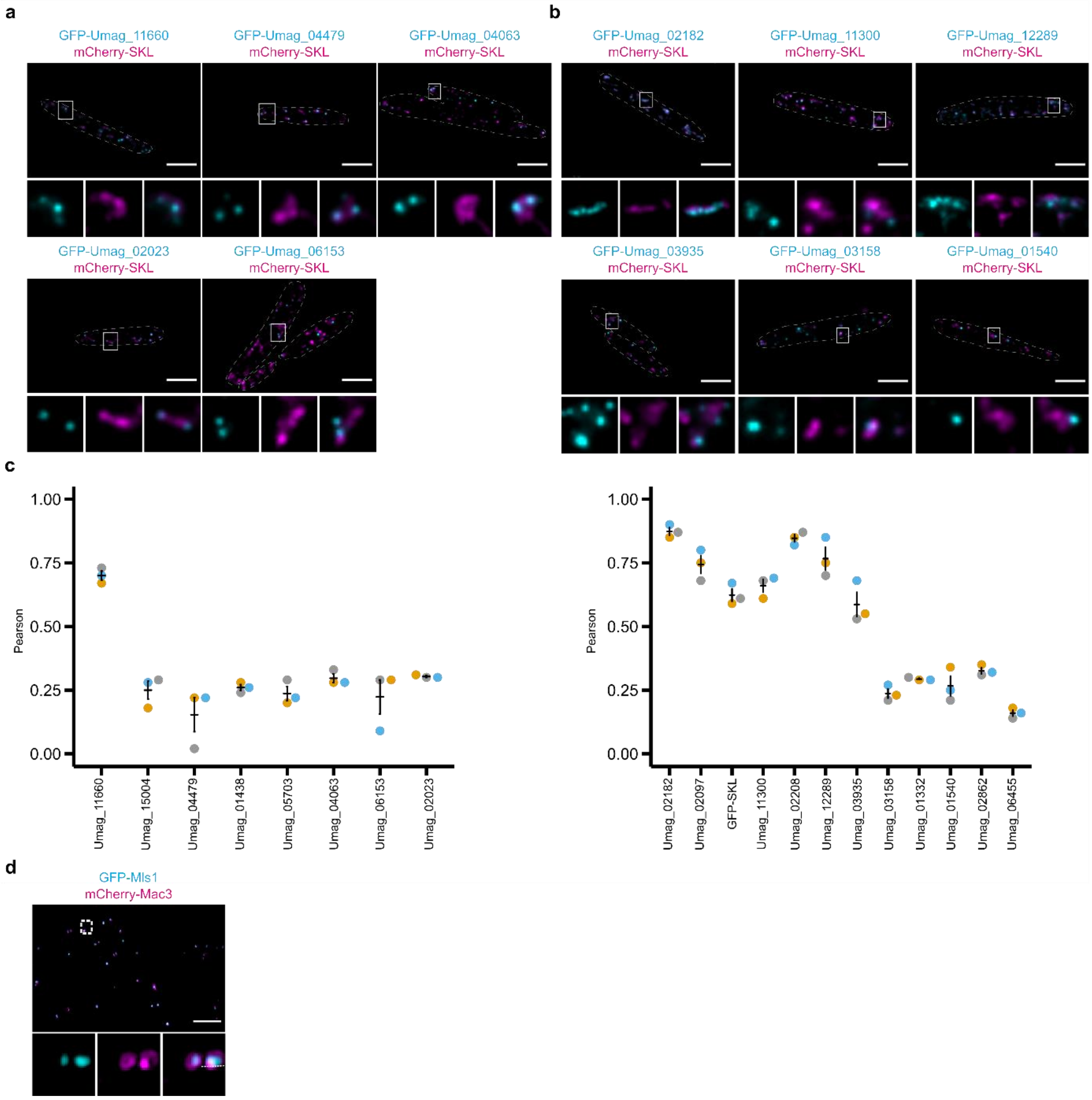

Figure S10 Analysis of candidate proteins with or without TIIV-like motif.
U. maydis strains expressing N-terminally GFP-tagged versions of candidate proteins (cyan) with TIIV like motifs **(a)** or without TIIV-like motifs **(b)** and the peroxisomal marker protein mCherry-SKL (magenta) were analyzed by epifluorescence microscopy. Full representative images are shown as overlays of the green and red channel. For insets single channels and merged channels are depicted. Umag numbers are gene identifiers accessible via the National Center for Biotechnology Information (NCBI). **c** Quantifications show Pearson's correlation coefficients of GFP and mCherry signals for indicated strains. Center line, mean; error bars; standard error of the mean. **d** Colocalization of indicated fusion proteins was analyzed by SIM. Organization of pictures as above. The associated graphs show the normalized fluorescence intensity of GFP and mCherry along the indicated lines. Plotted are the means of three replicates. Different colors denote single experiments. Center line, mean; error bars; standard error of the mean. Error bars on the data points denote the regression error, while the error bars on the column denote standard deviation of the mean.
Scale bars: 5 µm.

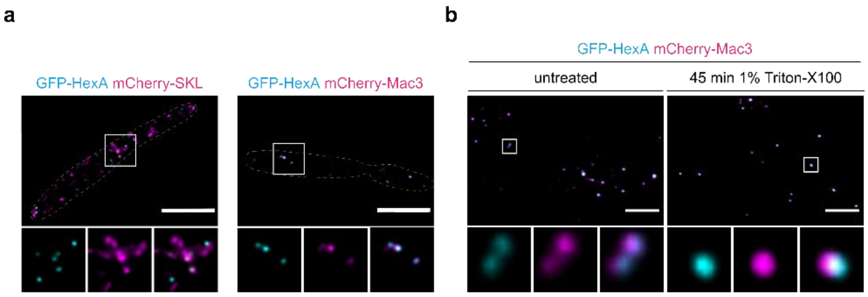

Figure S11 The Woronin body protein HexA colocalizes with Mac3.
a *U. maydis* strains expressing an N-terminally GFP-tagged version of *A. nidulans* HexA (cyan) and mCherry-SKL (magenta) or mCherry-Mac3 (magenta) were inspected by epifluorescence microscopy. Full images are shown as overlays of the green and red channel. For insets single channels and merged channels are depicted. **b** Crude organelle preparations of indicated strains were imaged by epifluorescence microscopy after incubation in lysis buffer supplemented with Triton X-100 (right). Preparations incubated in lysis buffer without Triton X-100 served as control (left). Organization of pictures as described above.
Scale bars: 5 µm.

References

Freitag, J. et al. Peroxisomes contribute to biosynthesis of extracellular glycolipids in fungi. Mol. Microbiol. 93, 24–36 (2014).

Becker, F. et al. Engineering *Ustilago maydis* for production of tailor-made mannosylerythritol lipids. Metab. Eng. Commun. 12, e00165 (2021).
