# Supplementary material for: Peroxisomal core structures segregate diverse metabolic pathways
Source: Nat Commun. 2025 Feb 20;16:1802. doi: 10.1038/s41467-025-57053-9 (PMC11842775; doi:10.1038/s41467-025-57053-9)
Supplement: Supplementary file 2 — Description of Additional Supplementary Files [file 41467_2025_57053_MOESM2_ESM.pdf]

Description of Additional Supplementary Files

File Name: Supplementary Data 1
Description: Candidates tested for core localization.

File Name: Supplementary Data 2
Description: Murine proteins with PTS1 and TIIV-like motif.

File Name: Supplementary Data 3
Description: Strains, plasmids and oligonucleotides.

File Name: Supplementary Data 4
Description: IJM File used to calculate Pearson values.

File Name: Supplementary Movie 1
Description: Time lapse movie of peroxisomal cores.
